# Derivation of Porcine Embryonic Stem-Like Cells from *In Vitro*-Produced Blastocyst-Stage Embryos

**DOI:** 10.1038/srep25838

**Published:** 2016-05-13

**Authors:** Dao-Rong Hou, Yong Jin, Xiao-Wei Nie, Man-Ling Zhang, Na Ta, Li-Hua Zhao, Ning Yang, Yuan Chen, Zhao-Qiang Wu, Hai-Bin Jiang, Yan-Ru Li, Qing-Yuan Sun, Yi-Fan Dai, Rong-Feng Li

**Affiliations:** 1 State Key Laboratory of Reproductive Medicine, Nanjing Medical University, Nanjing, Jiangsu, China; 2 Jiangsu Key Laboratory of Xenotransplantation, Nanjing Medical University, Nanjing, Jiangsu, China; 3 The Key Laboratory of the National Education Ministry for Mammalian Reproductive Biology and Biotechnology, Inner Mongolia University, Hohhot, Inner Mongolia, China; 4 State Key Laboratory of Reproductive Biology; Institute of Zoology; Chinese Academy of Sciences, Beijing, China

## Abstract

Efficient isolation of embryonic stem (ES) cells from pre-implantation porcine embryos has remained a challenge. Here, we describe the derivation of porcine embryonic stem-like cells (pESLCs) by seeding the isolated inner cell mass (ICM) from *in vitro*-produced porcine blastocyst into α-MEM with basic fibroblast growth factor (bFGF). The pESL cells kept the normal karyotype and displayed flatten clones, similar in phenotype to human embryonic stem cells (hES cells) and rodent epiblast stem cells. These cells exhibited alkaline phosphatase (AP) activity and expressed pluripotency markers such as *OCT4*, *NANOG*, *SOX2*, *SSEA-4*, *TRA-1-60*, and *TRA-1-81* as determined by both immunofluorescence and RT-PCR. Additionally, these cells formed embryoid body (EB), teratomas and also differentiated into 3 germ layers *in vitro* and *in vivo*. Microarray analysis showed the expression of the pluripotency markers, *PODXL*, *REX1*, *SOX2*, *KLF5* and *NR6A1*, was significantly higher compared with porcine embryonic fibroblasts (PEF), but expression of *OCT4*, *TBX3*, *REX1, LIN28A* and *DPPA5*, was lower compared to the whole blastocysts or ICM of blastocyst. Our results showed that porcine embryonic stem-like cells can be established from *in vitro*-produced blastocyst-stage embryos, which promote porcine naive ES cells to be established.

Embryonic stem (ES) cells are isolated from the ICM of preimplantation blastocyst stage embryos and are used extensively in both biomedical research and as a model to study early mammalian development^1^. The first ES cells were successfully derived directly from mouse blastocysts in 1981^2^,^3^. The ES cells were first isolated from non-human primate embryos in 1995^4^, human embryos in 1998^5^ and rats in 2008^6^. However, validated ES cells have not been generated from ungulate species so far, although ES like cell lines have been established from cattle^7^,^8^, rabbit^9^ and pig^10^,^11^,^12^,^13^,^14^,^15^,^16^,^17^,^18^.

Over the past about 25 years, many reports of porcine ES-like cell lines have been published^12^,^15^,^18^,^19^,^20^,^21^,^22^,^23^,^24^,^25^,^26^,^27^,^28^,^29^,^30^,^31^,^32^,^33^,^34^,^35^,^36^,^37^,^38^,^39^. However, none of these porcine ES-like cell lines satisfied the standards of real ES cells, even that of human ES cells. Attempts to create porcine ES cell lines have most often used *in vivo*-derived blastocysts^10^,^11^,^12^,^13^,^14^,^15^,^17^, which were shown to be superior to their *in vitro*-produced (IVP) counterparts^40^. However, the use of *in vivo*-derived blastocysts as starting material for isolation of ES cells is laborious and expensive. Considering the feasible availability and low cost, the porcine IVP blastocyst (BL) can be an alternative choice for ES cells culture^18^.

Production of these IVP embryos usually involves *in vitro*-maturation, *in vitro*-fertilization (IVF) and *in vitro*-embryonic development to blastocyst stage. Although IVP blastocysts may be altered in terms of cell metabolism, epigenetic status and constituent cell numbers, putative porcine ES cell lines could also be isolated from *in vitro*-produced intact blastocyst or ICM^23^,^26^. The pluripotency of porcine ICM is incontrovertible and previous studies have demonstrated that ICM can contribute to the embryo proper and form fetal chimeras following injection into blastocysts^19^,^41^, produce teratomas when they were injected into immunocompromised mice^42^. It was confirmed that ICM isolated from porcine blastocyst was superior to whole blastocyst regarding ES-like cell culture^27^. Both trypsin digestion and immunosurgery have been applied on ICM isolation and resulted in efficient derivation of porcine ES-like cells^15^,^27^,^39^. Recently, significant progress has been achieved regarding porcine ES-like cell derivation^23^,^32^,^43^,^44^,^45^. Telugu *et al*. derived leukemia inhibitory factor-dependent, so-called naive type, pluripotent stem cells from the ICM of porcine blastocysts by up-regulating expression of KLF4 and POU5F1 with lentivirus vector^45^. Haraguchi *et al*. generated self-renewing porcine embryonic stem-like cells from the ICM of porcine embryos by using inhibitors, CH99021 and PD184352^43^. Despite the endeavors and attempts mentioned above, the established porcine ES-like cell lines have failed to meet the criteria indicative of a true pluripotent state. The strategies used for these cell lines culture also have not resulted in consistent results so far. Most porcine ES-like cells derived from IVP embryo had not been thoroughly examined for their pluripotency and stem cell characteristics or cultured for more than a few passages *in vitro*. The differences in transcriptional profiling pattern between porcine ES-like cells and mouse ES cells or human ES cells have not been investigated.

In this study, we seeded porcine ICM isolated via immunosurgery method from day 6 and day 7 *in vitro*-produced blastocyst into α-MEM with bFGF. The putative porcine ES cells were derived. These porcine ES-like cells were confirmed to be pluripotent via a thorough investigation, including teratoma experiment and microarray analysis and had the characteristics very similar to human ES cells.

## Results

### Formation of Porcine ES -Like Cell Colonies from ICMs

**Figure 1:**
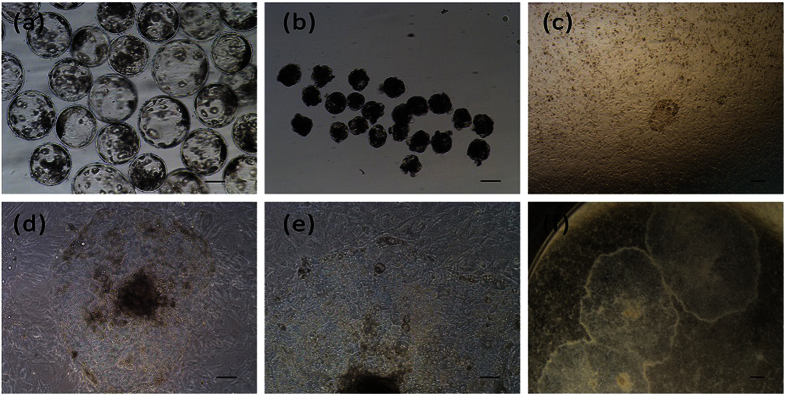
Derivation and morphology of pESL cells. (**a**) *In vitro* fertilized blastocysts at day 7. (**b**) Blastocysts after immunosurgery treatment. (**c**) Day 3 attached ICM. (**d**,**e**) The morphology of pESL cells colony at passage 2. (**f**) The morphology of pESL cells colony at passage 15. Scale bars = 100 um.

To get ICM-derived cell lines, we used an immunosurgery dissection technique to eliminate trophectoderm cells from day 6 and day 7 blastocysts (Fig. 1a) prior to seeding. To compare efficiency of MEF and STO feeder layer cells, total 103 ICMs (Fig. 1b) were placed on the STO or MEF feeder layer.

After 2–3 days culture, some of the ICMs attached to the feeder cell layers (Fig. 1c), the rest of the ICMs remained in suspension and were lost during the medium change. Cell proliferation was clearly observed in the attached ICMs and porcine ES-like cell colonies formed after another 3–4 days culture. The colonies were monolayer, epithelioid, densely packed with high nuclear to cytoplasmic ratios, distinct colony border and prominent nuclei (Fig. 1d–f) similar to human ES clones^5^. When the primary outgrowths reached 3–6 mm in diameter, they were mechanically cut into pieces and transferred to new culture dishes at day 10. Cells were then passaged every 7 days. New colonies emerged within 2–3 days of subculture, and the morphology of most new colonies was similar to the primary colonies.

ICMs had higher attachment rate (25.0%) and colony formation rate (21.2%) when STO cells were used as feeder cells, compared to that (11.8%, 3.9%) of MEF feeder cells. Total of 8 and 3 ES-like cell populations were obtained when STO and MEF were used as feeder layer, respectively. No colony derived from MEF feeder layer was passaged more than 10 times; However, 4 colonies were cultured more than 10 passages on STO feeder layer, and one of them was cultured up to passage 21, which was used for the further study.

### Expression of Pluripotency Markers in Porcine ES-like Cells

**Figure 2:**
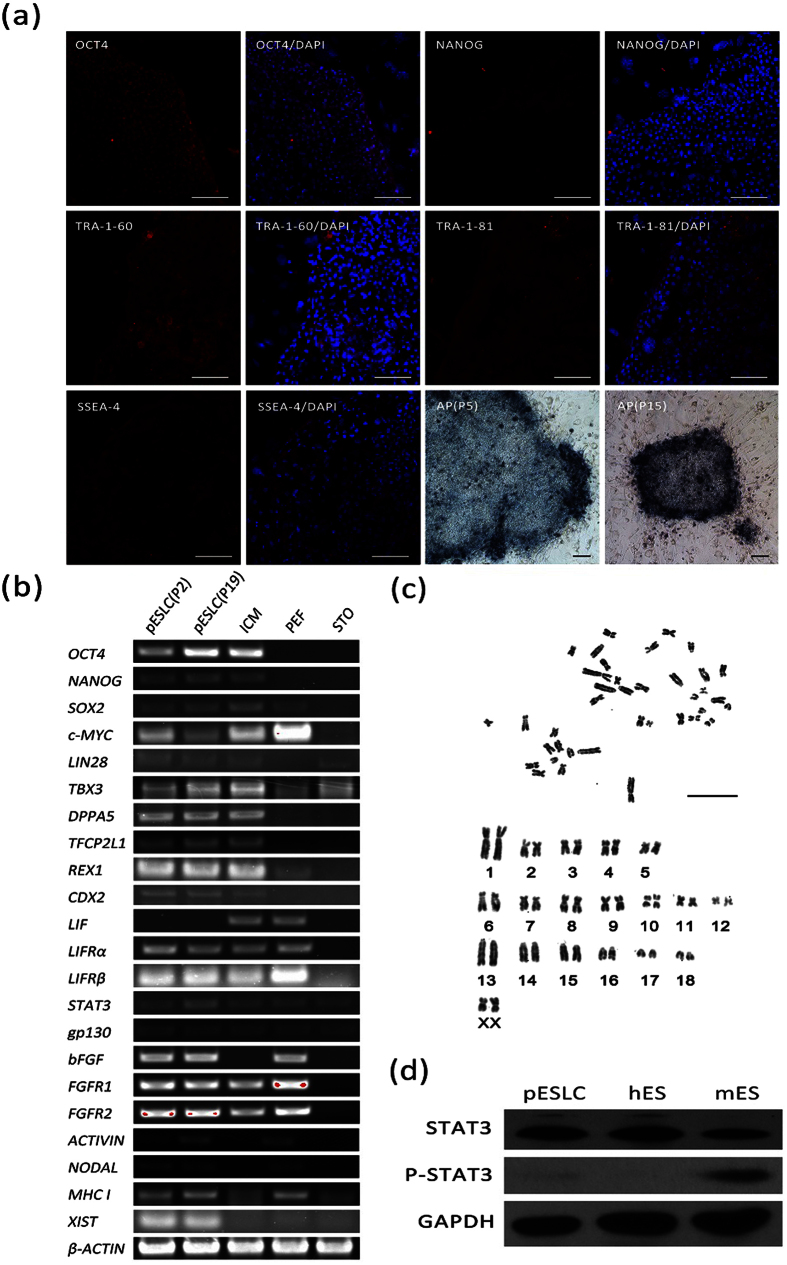
Characteristics of pESL cells. (**a**) The immunofluorescence staining of pluripotency markers OCT4, NANOG, SSEA-4, TRA-1-60, TRA-1-81 and alkaline phosphatase staining in pESLC colonies cultured on STO cells are shown. Scale bars = 100 um. (**b**) RT-PCR analysis of relative transcript concentrations of pluripotent and lineage-specific genes in pESL cells at passage 2 and passage 19, ICM, PEF, and STO. (**c**) Karyotype analysis of pESL cells at passage 7. Scale bars = 100 um. (**d**) Phosphorylation status of STAT3 in the pESLCs at passage 18. mES cells and hES cells were used as control. ICM: inner cell mass; PEF: porcine embryonic fibroblast cell; STO: feeder layer cell, SIM mouse thioguanne and ouabain resistant fibroblast cells.

Porcine ESLCs cultured *in vitro* over 20 passages, displayed normal morphology at passage 2 (Fig. 1d,e) and passage 15 (Fig. 1f) and showed a normal 38XX karyotype at passage 7 (Fig. 2c). The percentage of the cells with normal karyotype reached 70.5%. These cells at passage 5 and passage 15 displayed positive AP activities (Fig. 2a). Expression of ES cell markers, including *OCT4* (*POU5F1*), *NANOG, SSEA-4*, *TRA-1-60*, and *TRA-1-81*, were detected in pESLCs, porcine blasocysts and hESCs by immunofluorescence staining (Fig. 2a, Supplementary Fig. 1). However, *SSEA-1* expression had been detected in porcine blastocysts (Supplementary Fig. 1), but not in pESLCs and hESCs. The RNA transcripts of pluripotency markers, including *OCT4*, *NANOG*, *SOX2*, *c-MYC*, *LIN28*, *TBX3*, *DPPA5*, *TFCP2L1* and *REX1* were detected in pESLCs by RT-PCR (Fig. 2b). Except *LIF*, the ES related signal pathway genes, including *LIFRα*, *LIFRβ*, *STAT3*, *gp130*, *bFGF*, *FGFR1*, *FGFR2*, *ACTIVIN* and *NODAL*, were detected in pESLCs by RT-PCR (Fig. 2b) or Q-PCR (Supplementary Fig. 2). The *XIST* mRNA was detected in pESLCs, which is a characteristic associated with the inactivated X chromosome^46^; pESLCs were positive for MHC class I as assessed by RT-PCR. MHC class I expression is one of the hallmarks of the primed state^47^,^48^. Typical trophoblast marker gene *CDX2* was detected for lower expression in both pESLCs and porcine ICMs. To identify the genes bands derived from RT-PCR, *NANOG*, *TFCP2L1*, *TBX3, MHC I* and *c-MYC* were identified to be correct by sequencing (data not shown). Protein STAT3 phosphorylation is an important mark for the activated LIF signal pathway^49^. Western-blot analysis showed that protein STAT3 phosphorylation did not happen in pESLCs, as well as hES cells; on the contrary, protein STAT3 phosphorylation happened in mES cells (Fig. 2d).

### Differentiation of Porcine ES-like Cells *In Vitro* and *In Vivo*

**Figure 3:**
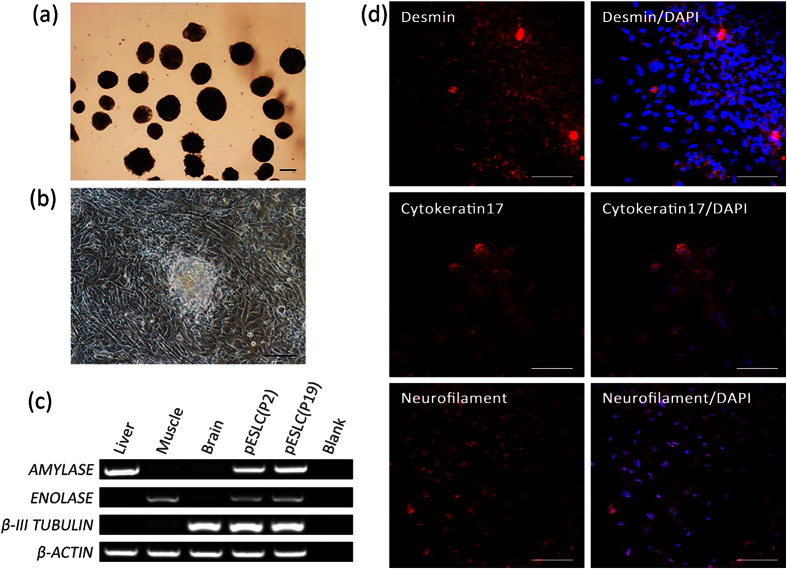
*In vitro* differentiation potential of pESL cells. (**a**) EB derived from pESLCs at passage 19 by hanging drop culture on no adhesive culture dishes for 10 days. (**b**) EB spread on dishes coated with gelatin and displayed distinct signs of differentiation. (**c**) Expression of the *β-III TUBULIN* (endoderm), *ENOLASE* (mesoderm), and *AMYLASE* (ectoderm) were detected in EB resulted from pESLCs at passage 2 and passage 19. Brain, liver and muscle tissues of Landrace pig were used as positive control. (**d**) Expression of differentiation marker Cytokeratin 17 (endoderm), Desmin (mesoderm) and Neurofilament (ectoderm) from differentiated pESL cells were confirmed by the immunocytochemistry analysis. Scale bar = 100 um.

The pESLCs at passage 2 and passage 19 that were cultured in the absence of bFGF on no adhesive culture dishes formed EB after 10 days of culture (Fig. 3a). When these EB were subsequently placed on dishes coated with gelatin and cultured in the same conditions for additional 3 days, they attached to the substratum, began to spread, and displayed distinct signs of differentiation (Fig. 3b). RT-PCR analysis of RNA isolated from EB derived from P2 and P19 pESLCs showed that the marker genes characteristic of the 3 germ layers (endoderm, *AMYLASE*; mesoderm, *ENNOLASE*; ectoderm, *TUBLIN*) were detected (Fig. 3c). The expressions of differentiation markers of 3 germ layers (endoderm, Cytokeratin 17; mesoderm, Desmin; ectoderm, Neurofilament) of differentiated cells from pESLCs were also detected through immunocytochemical analysis (Fig. 3d).

**Figure 4:**
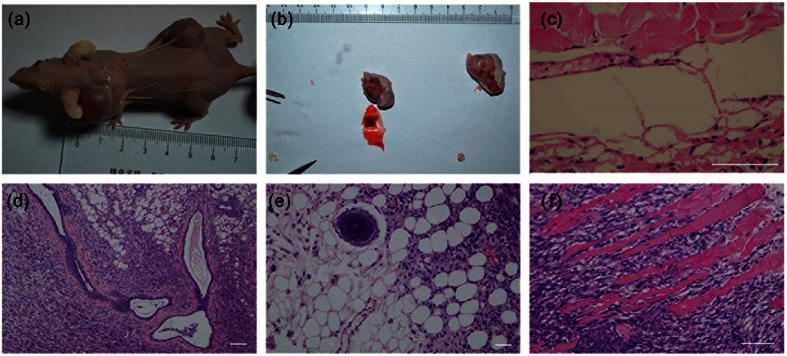
Teratoma formation from injected pESL cells. (**a**) Gross image of the mouse showing subcutaneous teratomas in the neck and dorsal flank regions. (**b**) Tumors removed from mouse (**a**) after 90 days injection. (**c**–**f**) Three germ layers present in teratomas derived from the pESL cell line at passage 21. The images of H&E-stained sections showing representative endoderm (**d**), branched glands and duct, mesoderm (**c**,**f**), striated muscle and smooth muscle and ectoderm lineage (**e**), neural rosette. Scale bar = 100 um.

To test *in vivo* differentiation properties of pESLCs, we injected pESLCs at passage 21 into four nude mice. One nude mouse died one month later. One mouse gave rise to two larger solid tumors (about 1 × 1.5 cm in size) in the neck and dorsal flanks (Fig. 4a) on histological examination; the other two mice both had a small solid tumor (about 3 × 4 mm in size) in the right dorsal flank after two months (Supplementary Fig. 3). The histological analysis of the teratomas sections showed that all three germ layers (Fig. 4c–f), including branched glands and duct (endoderm) (Fig. 4d), neural rosette (ectoderm) (Fig. 4e) and striated muscle and smooth muscle (mesoderm) (Fig. 4c,f) were observed, although the differentiated structures of identifiable tissue types were rarer. The teratomas were confirmed to be porcine and not murine origin by Real time-PCR and immunohistochemical analysis (Supplementary Fig. 4).

### Transcriptional Profiling of Porcine ES-like Cells by Microarray Analysis

**Figure 5:**
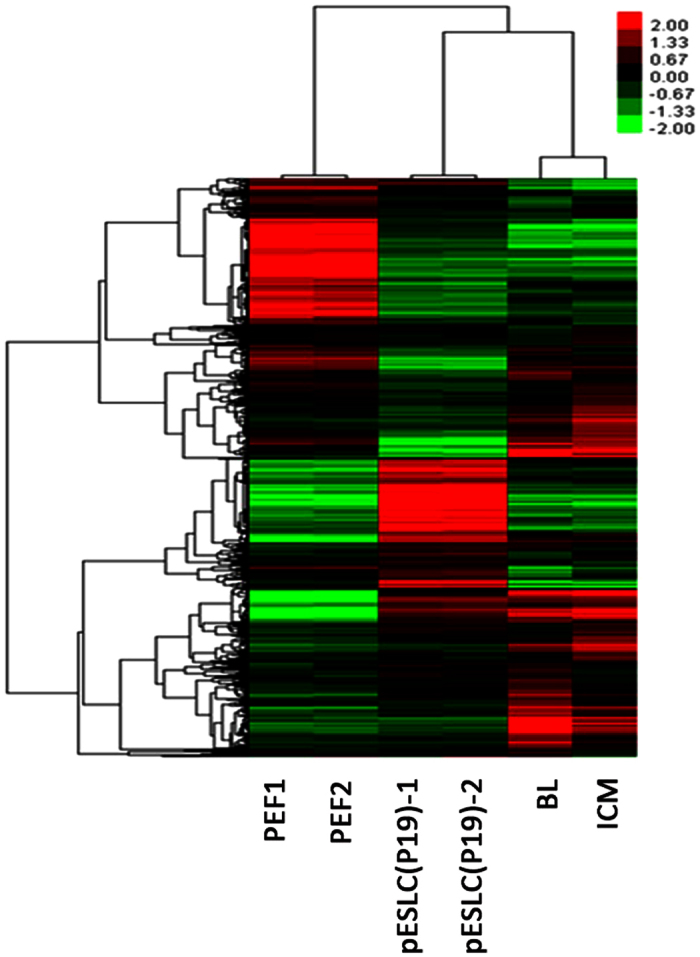
Gene expression profiling of pESL cells using Affymetrix microarrays data. Two-color heat map representation of globe gene expression data that derived from Affymetrix gene chip analysis of PEF1, PEF2, pESLC (P19), BL and ICM. Each row represents the expression of a single gene and columns indicate samples. PEF1, 2: Porcine embryonic fibroblast cell line PEF1 and PEF2 at passage 3; pESLC (P19)-1, pESLC (P19)-2: pESL cells at passage 19; BL: blastocysts; ICM: inner cell mass.

Microarray analysis were performed on pESLCs at passage 19, two PEF lines, ICMs, and BL served as negative or positive control. The global gene expression profiles of the pES-like cell line were compared with two PEF lines, ICM and BL using the Affymetrix Gene Chip Porcine Genome Array. Three groups could be identified in the heat map (Fig. 5). Hierarchical clustering result showed the global gene expression pattern of pESLCs is more similar to that of ICM and BL than to PEFs.

**Figure 6:**
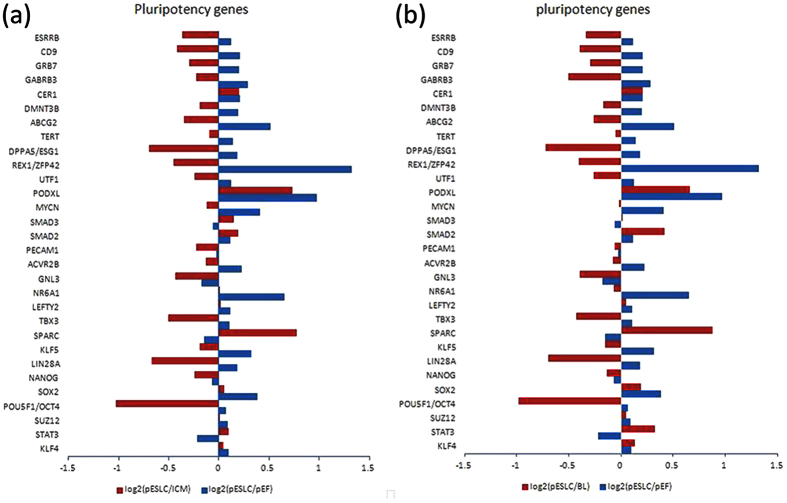
Pluripotency gene expression analysis of pESL cells was performed using Affymetrix microarrays data. (**a**) Log2-fold change in pluripotency genes expression in pESLCs compared with ICMs (red bars) and PEFs (blue bars). (**b**) Log2-fold change in pluripotency gene expression in pESLCs compared with *in vitro* fertilized d7 blastocysts (red bars) and PEFs (blue bars).

Also, the log2-fold changes in expression levels of pluripotency genes in pESL cells compared with blastocysts and PEFs are shown in Fig. 6. Most of pluripotency genes were expressed in pESLCs at lower levels compared with that in ICM and blastocysts. These genes include *OCT4, NANOG, LIN28A, REX1* (*ZFP42*)*, KLF5, TBX3, DPPA5* (*Esg1*)*, TERT, DMNT3B, GNL3, ACVR2B, PECAM1, UTF1, ESRRB* and so on. However, expression of the overwhelming majority of the pluripotency genes, such as *OCT4, SOX2, NR6A1, PODXL, KLF4, KLF5, ESRRB, REX1, TERT, LEFTY2, ABCG2* and *CER1*, was significantly higher in pESLCs compared with that in PEF. Interestingly, some pluripotency genes such as *KLF4, SOX2, STAT3, SMAD2, SMAD3, PODAL*, and *CER1*, were expressed at a slightly higher levels in pESLCs compared with ICM and blastocysts. Based on the microarray data, the expression of the important stem cells transcription factors *OCT4, LIN28A, REX1, TBX3*, and *DPPA5* are much higher in ICM than pESLCs (Supplementary Table 1). As trophectoderm removed, the value of log2 (pESLC/ICM) was lower than that of log2 (pESLC/BL).

### Comparison of Transcriptional Profiling among Porcine ES-like Cells and Other Species ES Cells

**Figure 7:**
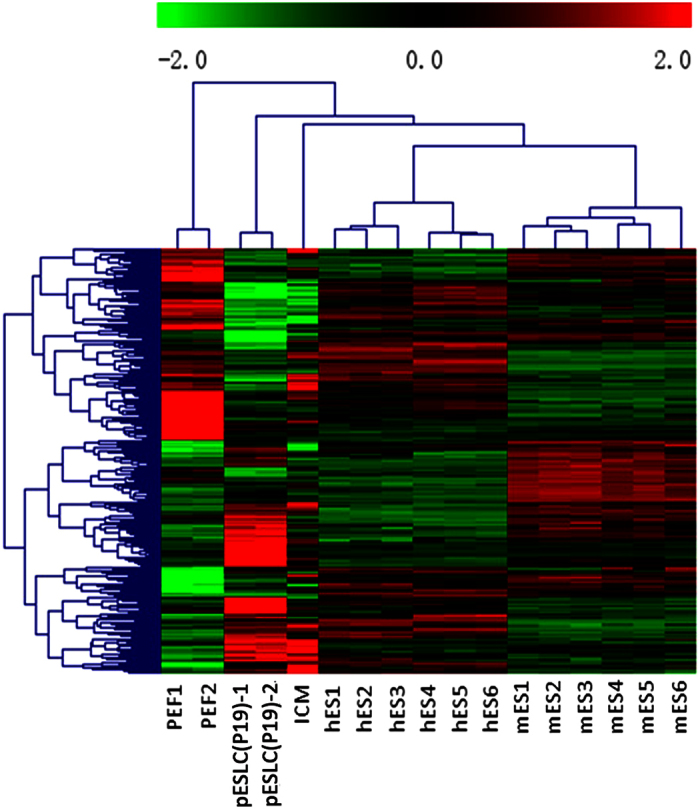
Hierarchical cluster analysis of the orthologous genes expression profile of pESLCs. Two-color heat map representation of the orthologous genes expression data of PEF, pESLCs, hES cells and mES cells. Each row represents the expression of a single gene and columns indicate samples; PEF1, 2: Porcine embryonic fibroblast cell line PEF1 and PEF2 at passage 3; pESLC(P19)-1, pESLC(P19)-2: pESL cells at passage 19; hES 1, 2 and 3: hES cell line HUES6; hES 4, 5 and 6: hES cell line H9; mES 1, 2 and 3: mES cell line R1; mES 4, 5 and 6: mES cell line J1.

Microarray analysis was performed to compare the patterns of gene expression exhibited by pESLCs, PEF, porcine ICM cells, mouse ES cells and human ES cells. A hierarchical cluster analysis of 256 orthologous genes in pig, mouse and human was performed based on the gene-expression profiles (Supplementary Table 2). This analysis showed that the gene-expression profile of the pES-like cell line was significantly different from the somatic cells (PEF). However, the expression profiles of pESLCs were very similar firstly to that of porcine ICM cells and then to that of hES cells and mES cells (Fig. 7). These results revealed pES-like cells’ stem cell characters.

## Discussion

The ES cells hold great promise for therapeutic use and represent a unique tool for investigating the process of self-renewal and differentiation. The number of papers describing porcine ESCs is less than that of mouse and human ESCs, and none of the porcine cell lines described in the literature satisfy all the criteria required for a formally correct definition of embryonic stem cells based on mouse ESCs standards. Moreover, the technologies for the derivation and genetic manipulation of murine ES cells are well established, and germline chimeras have only been produced in the mouse^50^, rat^6^ and chicken, although the latter were passaged for a maximum of only three times^51^. Attempts have been made to develop ES cells in the pig, but this has met with limited success. To our knowledge, only Hochereau-de Reviers MT *et al*. obtained teratomas from non-induced putative porcine ES cells^13^; moreover, there have been only two reports claiming the production of chimaeric pigs^15^,^23^, but no reports of germline transmission.

In this study, we derived putative porcine ES cells from ICMs of day 6 and day 7 *in vitro*-produced blastocyst. Guangyun Tan *et al*. demonstrated that the cells from the day 7 porcine embryos are pluripotent but the cells from the day 9 embryos lose pluripotency, and ES-like cells could not be isolated from the day 5 embryos; The expression of the pluripotency factors (*OCT4*, *NANOG*, *SOX2*, and *c-MYC*) increased from day 5 to day 7, whereas the expression decreased from day 7 to day 9; the number of ICM cells from day 7 porcine blastocysts is more than that of ICM cells from day 5 and day 9 blastocysts^44^. Thus, the possibility of establishing stable pluripotent cell lines using day 6 and day 7 blastocysts is much greater than with day 5 and day 9 blastocysts. These results fall in line with work by Brevini T. A. *et al*.^52^. The culture of intact porcine blastocysts is not suitable as this approach results in attachment and growth of the trophoblast and primitive endoderm while the embryoblast does not grow and instead degenerates or differentiates^38^. We compared the morphology of our pESLCs with those of cell lines derived from whole blastocysts, which contain multiple lipid droplets (data not shown). Irena Vackova *et al*. also claimed that the cell lines derived from whole blastocysts were trophectoderm cells^53^. In this study, about 30% of colonies derived from ICMs isolated by immunosurgery dissection were abundant for lipid droplets, it was most likely that trophectoderm cells were not eliminated completely from blastocysts. These lipid abundant cell lines could not be cultured beyond 10 passages in this culture system.

Immunofluorescence or RT-PCR detected the expression of pluripotency markers, such as AP, *OCT4*, *NANOG*, *SOX2*, *TBX3*, *REX1*, *DPPA5*, *SSEA-4*, *TRA-1-60*, and *TRA-1-81* etc. in the cell line derived in this study. It was reported that expression of Rex1 is limited in the ICM of blastocysts in mouse, subsequently down-regulated during the later stages of differentiation in the epiblast and primitive ectoderm (PrE)^54^. However, Rex1 expression is very low or undetectable in pluripotent EpiSCs (epiblast stem cells, post-implantation epiblast-derived stem cells)^55^,^56^. The ES cell-specific surface antigen SSEA family in pESLCs seems to have similar expression pattern with hES cells and some porcine pluripotent stem cells. The pESLCs shown the expression of SSEA4, rather than SSEA1. It is well known that SSEA1 is expressed on mES cells, and it is low or absent in hES cells, and a series of antigen *SSEA*-*3* and *SSEA*-*4*, are displayed on the human ES cells^57^. Some reports claimed that porcine pluripotent stem cells are negative for SSEA-1^58^; however, Telugu B. P. *et al*.^45^, Ivan Vassiliev *et al*.^23^ and Fujishiro S. H. *et al*.^59^ did find the *SSEA-1* expression in their putative porcine ES cells and porcine iPS cells, respectively.

The pESLCs formed teratomas when injected into nude mice, and differentiated into 3 germ layers *in vivo*. Additionally, these cells generated flattened clones and kept the normal karyotype. The cell line survived repeated passage and continued to maintain their morphology and karyotype for up to 21 passages, suggesting that these lines are stable. Hence, the pES-like cells derived in this study are pluripotent, and generation of pES cell lines from ICMs of IVP embryos is feasible. These results also indicated that the use of medium containing a combination of bFGF, EGF, Activin-a, ITS, and KOSR is effective to promote attachment, outgrowth and expansion of porcine ICMs.

Porcine ES cells should be the result of a selection and adaptation process of ICMs to the culture environment. The past attempts to establish ungulate cell lines that fulfill all the criteria of ES cells have not been successful due, in part, to the inability to optimize culture conditions. The pES cells culture systems that are generally used based on mouse ES cell culture methods^60^ are not optimal for porcine ES cell line. Mouse and pig are substantially different in early embryonic development. The mouse has a short invasive pre-implantation period while the pig has a longer non-invasive pre-implantation period that is characterized by rapidly dividing trophectoderm cells and a rather quiescent ICM cells^30^. Maintenance of mouse ES cells depends on the presence of leukemia inhibitory factor (LIF)^30^. Most previous reports have shown no beneficial effect of the addition of LIF to the maintenance of porcine stem-like cells in an undifferentiated state^13^,^29^,^30^,^38^,^39^,^61^. Thus, LIF signal seemed not to be critical for maintaining porcine primed ES cells^62^. Although mRNA expression of LIF pathway (*STAT3*, *gp130*, *LIFRα* and *LIFRβ*), FGF pathway (*bFGF*, *FGFR1*, *FGFR2*) and Activin pathway (*ACTIVIN* and *NODAL*) related pluripotency genes were detected in the porcine ES-like cell lines derived in this study, the phosphorylation status of STAT3 protein was not detected in pESLCs, which indicated that LIF signal path was not activated in pESLCs. Pant and Keefer demonstrated that the bovine ICM and its primary outgrowths do have the LIF receptor and gp130 signal transducer^63^. It may be that, similar to human ES cells, activation of this pathway in ungulate pluripotent cells will induce differentiation instead of proliferation; and LIF results in a stronger stimulation of the differentiation-inducing mitogen-activated protein kinase kinase/ extracellular regulated kinase MEK/ERK pathway than the STAT3 pathway^64^. However, Seiki Haraguchi *et al*.^43^ and Telugu *et al*.^45^ established LIF-dependent porcine naive-like stem cell lines with three-dimensional domed colony morphology via supplementing LIF and small molecular inhibitors into the culture medium, which are specific for FGF signal pathway and GSK3 inhibition. Their results demonstrated that only LIF is not enough for LIF-dependent porcine ES cells culture, combination with small molecular inhibitors will be necessary. Based on above observation and analysis, the LIF might be critical for porcine naive ES cells, not for porcine primed ES cells.

The feeder cell layer is one of the most important factors affecting ES cell culture. It serves as an attachment matrix for cells and can secrete some kinds of cytokines that may stimulate ES cell growth and inhibit their differentiation^27^. The various feeder cell types have been tested for establishing stably expandable pES-like cells^24^,^28^,^33^. Among these feeder cell types, the STO and MEF were investigated in this study. Some papers showed that MEFs are better for culturing pES-like cells than STO cells^28^. Talbot *et al*. demonstrated that STO cells were required for the survival of porcine and bovine epiblast cells in primary culture^38^,^65^. STO feeder cells have been used successfully not only for most porcine ES-like cells derivation^12^,^15^,^19^,^20^,^25^,^28^,^31^,^34^,^37^,^66^,^67^,^68^,^69^, but also for mouse and human ES cell derivation^3^,^70^. STO cells were further confirmed to be effective for porcine primed ES cell culture in this study.

At present, new resources such as gene banks and microarrays can be made in animal stem cell biology, which would be in favor of identifying specific pluripotency markers. Microarray data have been used to compare and identify unique expression patterns of transcripts in human and mouse ES cells^71^,^72^,^73^. In this study, via comparing the pES-like cells with hES cells and mES cells regarding the globe transcriptional profiling, we confirmed the stem cell pattern of the derived pES-like cells. The expression pattern of pES-like cells had the most similarity to that of porcine ICM cells and then to that of hES cells and mES cells, which was consistent with Wu *et al*.^58^, but not with Jin-Kyu Park *et al*.^32^. We also found that the expression of the important stem cells transcription factors, such as *OCT4*, *LIN28A*, *REX1*, *TBX3*, and *DPPA5*, are much higher in porcine ICM than pESLCs. *OCT4*, *SOX2*, *c-MYC* and *KLF4*, which were screened by Yamanaka S and used to generate mouse iPS^74^ and human iPS^75^, are the most commonly used on porcine pluripotent stem cell induction^58^,^59^,^76^. However, the effect of the new transcription factors combination (*OCT4*, *LIN28A*, *REX1*, *TBX3*, and *DPPA5*) found in this study on porcine pluripotent stem cell induction needs to be investigated. Compared to human and mouse, the different developmental pattern of the pig embryo may contribute to clarification as to why the porcine embryonic stem cells are harder to establish than human and mouse embryonic stem cells.

## Materials and Methods

### Animal Care and Use

All experiments with animals were approved by the Institutional Animal Care and Use Committee (IACUC) of the Nanjing Medical University and the methods were carried out in “accordance” with the approved guidelines.

### Chemicals

All chemicals were purchased from Sigma (St. Louis, MO) unless otherwise indicated.

### Oocyte Collection and *In Vitro* Maturation (IVM)

Ovaries were retrieved from prepubertal gilts at a local abattoir, the Slaughterhouse of Meat Processing Factory in Nanjing, and transported to the laboratory in physiological saline (0.9% NaCl) at 30–35 °C within 2 h. Oocyte collection and *in vitro* maturation was as described previously^77^.

### Embryo Production

Fresh Landrace boar semen was purchased from Pig Breeding Farm of Jurong Agriculture School and washed twice with 10 ml sperm wash medium (Dulbecco’s PBS supplemented with 0.1% BSA). Spermatozoa were then diluted in modified Tris- buffered medium (mTBM) containing 113.1 mM NaCl, 3.0 mM KCl, 7.5 mM CaCl_2_.2H_2_O, 20.0 mM Tris, 11.0 mM glucose, 5.0 mM sodium pyruvate, and 0.1% (w/v) BSA to give a concentration of 1 × 10^6 ^sperm/ml. After 42–44 h of maturation culture, cumulus oocyte complexes (COCs) were transferred to TL-HEPES containing 0.01% PVA (w/v) and 0.1% hyaluronidase (w/v), vortexed to remove the cumulus cells. Subsequently oocytes with the first polar body were selected for IVF. Oocytes were gently washed 6 times with mTBM, every 40 oocytes were transferred to 100 ul mTBM drop, and sperm was added to the COCs at a final concentration of 5 × 10^5 ^sperm/ml. After 4–6 h, oocytes were gently washed 6 times to eliminate sperm adhering to the zona pellucida. Finally, embryos were cultured in groups of 50–70 in PZM-3 medium (108.0 mM NaCl, 10.0 mM KCl, 0.35 mM KH_2_PO_4_, 0.4 mM MgSO_4_·7H_2_O, 25.07 mM NaHCO_3_, 0.2 mM Na-pyruvate, 2.0 mM Ca(lactate)_2_·5H_2_O, 1.0 mM glutamine, 5.0 mM hypotaurine, 20 ml/L Eagle’s basal medium amino acid solution, 10 ml/L modified Eagle’s medium amino acid solution, 0.05 mg/ml gentamicin, 3 mg/ml BSA) covered with mineral oil, at 38.5 °C, 7% O_2_, 5% CO_2_, 88% N_2_ in 100% humidity.

### ICM Isolation and Culture

After 7 days development culture, IVF embryos were cultured to the early blastocysts stage. The zona pellucida of blastocysts were removed in 0.5% pronase solution for 10 seconds. Blastocysts were gently washed 6 times in pESLCs medium to eliminate residual pronase. Subsequently, to isolate ICM cells from the trophectoderm, blastocysts were incubated in 10% Rabbit anti-Porcine Serum for 30 min and 10% Guinea Pig Complement for 30 min. ICMs were isolated from lysed trophoblast cells by pipetting and washed through several pESLCs medium drops to avoid culture oil carry-overs and encourage better attachment.

Isolated ICMs were seeded in a 4-well plate (Thermo Scientific, USA) onto a monolayer of mitomycin C-inactivated mouse STO (SIM mouse embryo derived thioguanine and ouabain resistant cell line, ATCC) and MEF (mouse embryo fibroblast line, ATCC) with 0.5 ml medium. Both STO and MEF were cultured in high glucose DMEM, supplemented with 10% fetal bovine serum (FBS), inactivated by 10 μg/ml mitomycin-C for 2.5 h, re-suspended in culture medium and seeded at a density of 1.75 × 10^5^ and 1 × 10^5 ^cell/well respectively in 4-well dishes, pre-coated with 0.1% gelatin. The pESLCs medium consisted of α-MEM supplemented with 20% KOSR, 20 ng/ml bFGF, 20 ng/ml EGF, 10 ng/ml Activin-a, 1% Insulin-Transferrin-Selenium, 1 mM MEM non-essential amino acid, 55 μM β2-Mercaptoethanol. The ICMs were cultured at 38.5 °C in a 5% CO_2_ atmosphere. The cultured medium was changed two days after the blastocysts were transferred to the ESLCs medium and 400 ul per well of medium was changed every day following the first 2 days.

### Karyotyping

Karyotyping was performed at passage 7 according to a method as described by Qiang Liu *et al*.^77^. Briefly, pESLCs colonies were cultured for 7 days after passage and the medium then was replaced with fresh pESLCs medium containing 20 ng/ml colcemid for 1 h at 38.5 °C in a 5% CO_2_ atmosphere. Colonies of pESLCs were cut into small pieces and digested in 0.25% trypsin for 5 min and resuspended in a 0.56% potassium chloride solution, incubated at 37 °C for 15 min, and fixed in freshly prepared 2 ml methanol : acetic acid (3:1) at 4 °C for 3 min, followed by 3 fixation steps, each for 20 min. The cell pellets were resuspended in fixation solution, and one drop of fixation medium with cells was placed on each pre-cooled clean glass slide. Images of chromosome spread were captured using the Olympus microscope (1–71, Olympus, Japan).

### Alkaline Phosphatase Staining and Immunocytochemistry

Alkaline phosphatase (AP) activity of the pESLCs at passage 5 and passage 15 was detected with an AP detection kit (S3771, Promega, USA) according to the manufacturer’s instructions. Briefly, cells were washed with Dulbecco’s phosphate- Buffered Saline (DPBS), then fixed with 4% paraformaldehyde for 10 min at room temperature. After rinsing with PBS, fixed cells were stained with alkaline phosphatase buffer containing nitro blue NBT (tetrazolium chloride) and BCIP (5-bromo-4-chloro-3-indolyl phosphate toluidine salt) stock solution for 30 min at room temperature in the dark. The cells were washed with DPBS before observation.

For immunofluorescent staining, day 6 and day 7 porcine blastocysts, pESLCs at passage 16, hESCs at passage 39 (cell line hES-8, kindly provided by Dr. Ge Lin of Central South University) and the differentiated cells resulted from pESLCs were rinsed in DPBS for 5 min at room temperature, then fixed in 4% paraformaldehyde in DPBS for 5 (for blastocysts) or 10 min (for pESLCs, hESCs and the differentiated cells). The blastocysts and hESCs served as control for pESLCs. After washing in DPBS, cells were permeabilized (for intracellular markers only) in 1% Triton X-100 for 1 h and eventually blocked in 10% goat serum (blocking solution) for 1 h at room temperature to block nonspecific binding. Primary antibodies applied were OCT4 (1:100, SC-9081, Santa Cruz Biotechnology, USA), NANOG (1:100, MABD24, Chemicon, Germany, Germany), SSEA-1 (1:100, MAB4301, Milllipore , Germany), SSEA-4 (1:100, MAB4304, Milllipore, Germany), Tra-1-60 (1:100, MAB4360, Milllipore, Germany), Tar-1-81 (1:100, MAB4381, Milllipore, Germany), Neurofilament (1:200, MAB1615, Milllipore, Germany), Desmin (1:200, MAB3430, Millipore, Germany) and Cytokeratin 17 (1:200, MAB1625, Millipore, Germany). All cells were treated with primary antibodies in the blocking solution for 24 h at 4 °C. Subsequently, cells were rinsed in 1× DPBS and incubated with secondary antibodies in the blocking solution for 1 h at 4 °C. Secondary antibodies used were Alexa Fluor 546 goat anti-mouse lgG (H + L) (1:200, A11030, Invitrogen, USA), donkey anti-rabbit-CY3 (1:200, A21206, Molecular Probes, USA) and FITC conjugate Goat Anti-Mouse IgG & IgM Antibody (1:200, AP130F, Millipore, Germany). The cells were then washed and stained with DAPI (Fluoroshield with DAPI, F6057, Gibco, USA). Stained cells were examined using confocal images system (710, Zeiss, Germany). The image acquisition, analysis, and processing were standardized within each experiment.

### Reverse Transcription and Polymerase Chain Reaction

To analyze the relative abundance of mRNA transcripts of pluripotent and differential genes, pESLCs were collected at passage 2 and passage19. Total mRNA was extracted from pESLCs (P2), pESLCs (P19) and corresponding EB, ICMs, PEF (P3) and tissues (brain, liver and muscle) of Landrace pig using the TRIzol Reagent (B5704-1,Takara, Dalian, China) according to the manufacturer’s instructions, followed by treatment with DNase I (2212, Takara, Dalian, China) according to the manufacturer’s protocol. RNA quality and quantity were determined using a Spectro-photometer (NanoDrop 2000c, Thermo Scientific, USA). cDNA was synthesized immediately after using PrimeScript^TM^ RT reagent Kit (RR037A, Takara, Dalian, China) following the manual supplied by the manufacturer. RNA from ICM of porcine day 7 blastocysts and tissues (brain, liver and muscle) of Landrace pig served as positive control, RNA from STO feeder cell and PEF served as negative control. Q-PCR was performed using Light Cycler PCR QC Kit (Roche, Switzerland) and the 7300 Real-Time PCR System (LC96, Roche, Switzerland). RT-PCR was performed using Veriti 96 cell Thermal Cycler (Applied Biosystems, USA). *β-ACTIN* served as positive control. PCR primers were listed in Supplementary Table 3. The following PCR conditions were used: 30–35 cycles including denaturation at 94 °C for 30 s, annealing for 30 s and elongation at 72 °C for 30 s, finalized by elongation at 72 °C for 10 min and incubation at 4 °C. PCR products were run on a 1% agarose gel and imaged using a ChemiDoc XRS+ Molecular Imager (Bio-Rad, USA) after staining with ethidium bromide for 5 min.

### Western Blotting

Western blotting was performed to detected phosphorylation of STAT3 in pESLCs at passage 18. The mES cells at passage 26 (cell line R1-E, kindly provided by Dr. Xueling Li of Inner Mongolia University) and hES cells at passage 28 (cell line hES-8) were used to be positive and negative control. Western blotting was performed according to methods described by Seki Haraguchi *et al*.^43^. Briefly, 30 ug of total cell lysate from pESLCs, mES cells and hES cells were first resolved on 12% SDS-PAGE gels. Protein bands were transferred electrophoretically onto PVDF membranes (Millipore) and incubated in blocking buffer (TBST: 10 mM Tris-HCl, 150 mM NaCl, 0.05% Tween, pH 7.5) containing 5% nonfat milk and 1% BSA (Sigma) for 1 h. Membranes were then exposed to primary antibodies anti-STAT3 (1:1000, 4094P, Cell Signaling Technology, USA), anti-phospho-STAT3 (Tyr705) (1:1000, 9145S, Cell Signaling Technology, USA) and anti-GAPDH (H-12) (1:1000, sc-166574, Santa Cruz Biotechnology, USA) in blocking buffer for 2 h at room temperature, washed for 3 times with TBST, then incubated with goat anti-rabbit IgG-HRP secondary antibody (1:5000, sc-2004, Santa Cruz Biotechnology, USA) or goat anti-mouse IgG-HRP secondary antibody (1:5000, sc-2005, Santa Cruz Biotechnology, USA). After further washing for 3 times in TBST, the blots were imaged by ChemiDoc XRS+ Molecular Imager (Bio-Rad, USA).

### Embryoid Body Formation

For EB formation, colonies of pESLCs at passage 2 and passage 19 were cut into small pieces and cultured in hanging drop in pESLCs medium without bFGF for 7 days. The EB was subsequently placed on dishes coated with gelatin and cultured in the same conditions for additional 7 days.

### Teratoma Formation

Teratomas were derived by injecting 5 × 10^6^ pES-like cells at passage 21 from 16 4-well plates into subcutaneous tissue at the neck and dorsal flanks of four 6-week-old athymic immunocompromised nude mice (BALB/C nude mouse, Charles River, Beijing, China). Three months post injection, tumors were derived and fixed in Bouin’s solution, which included saturated picric acid, formaldehyde, and glacial acetic acid. Tumors were then paraffin-embedded, sectioned and stained using hematoxylin and eosin. The DNA extraction from paraffin-embedded tissue was performed following the instruction of QIAamp DNA FFPE Tissue Kit (56404, Qiagen, Germany). The real-time PCR to detect porcine DNA of paraffin-embedded sections was performed following the instruction of TAKARA Real-Time PCR porcine DNA Detection kit (RR921, TaKaRa, Dalian, China). For immune- histochemical analysis, the paraffin sections were dewaxed first, then heat-mediated antigen retrieval was performed by microwaving sections for 20 min in 10 mM Sodium Citrate, pH 6.0. Sections were allowed to cool for 15 min, followed by a brief wash in deionized water, and rinsed twice in PBS. Sections were incubated for 30 min in 5% goat serum in DPBS containing 0.1% Tween and 0.5% BSA. The sections were incubated overnight at 4 °C with primary antibody Mouse anti-Porcine IgM Antibody (GTX75506, GeneTex, USA) at the appropriate dilution. The second antibody Dako REAL EnVisio Detection System (K5007, DAKO, Denmark) was used to detect porcine IgM staining.

### Transcriptional profiling by microarray

For Microarray analysis, total RNA from pESLCs at passage 19, two PEF lines at passage 3, porcine ICMs and porcine BL (whole blastocysts) was extracted using the TRIzol Reagent (B5704-1, TaKaRa, Dalian, China) based on the manufacturer’s instructions. The PEF served as negative control and ICMs and *in vitro*-produced day 6 blastocysts served as positive control. The pESLCs were used to perform a whole-genome expression profiles (microarray analysis) using the Affymetrix Gene Chip Porcine Genome Array (Porcine Gene 1.0 ST Array), which are based on the Sscrofa9 (susScr2) database, contains 394580 probe sets, that represent over 27559 transcripts. The Euclidean distance was used as the cluster method for heatmap-making.

### Comparison of Transcriptional Profiles among Porcine ES-like Cells and Other Species ES Cells

Human ES cells and mouse ES cells expression data information (hES cells: GSM628197-9, GSM525424-6; mES cells: GSM64922, GSM64924, GSM64926, GSM72622, GSM72624 and GSM72626) were downloaded from NCBI GEO and used to compare global gene expression with the pES-like cells. Porcine ICMs and PEF served as positive and negative control respectively. The signal ratios of orthologous genes were used to perform hierarchical clustering (conditioned tree) with Gene Spring 10.0.

## Additional Information

**How to cite this article**: Hou, D.R. *et al*. Derivation of Porcine Embryonic Stem-Like Cells from *In Vitro*-Produced Blastocyst-Stage Embryos. *Sci. Rep*. **6**, 25838; doi: 10.1038/srep25838 (2016).

## Supplementary Material

Supplementary Information

Supplementary Table S1

Supplementary Table S2
