# Supplementary material for: Derivation of Porcine Embryonic Stem-Like Cells from In Vitro-Produced Blastocyst-Stage Embryos
Source: Sci Rep. 2016 May 13;6:25838. doi: 10.1038/srep25838 (PMC4865852; doi:10.1038/srep25838)
Supplement: Supplementary Information [file srep25838-s1.doc]

**Supplementary information for**

**Derivation of porcine** **Embryonic Stem-Like Cells from In Vitro-Produced Blastocyst-Stage Embryos**

Dao-Rong Hou, Yong Jin, Xiao-Wei Nie, Man-Ling Zhang, Na Ta, Li-Hua Zhao, NingYang, Yuan Chen, Zhao-Qiang Wu, Hai-Bin Jiang, Yan-Ru Li, Qing-Yuan Sun, Yi-fan Dai, Rong-Feng Li

**This file includes:**

Supplementary Figures 1 – 4

Supplementary Tables 1 – 3

**Supplementary Figures 3**

**Supplementary Tables 8**

**Supplementary Figures**

**Supplementary Figure 1**

**
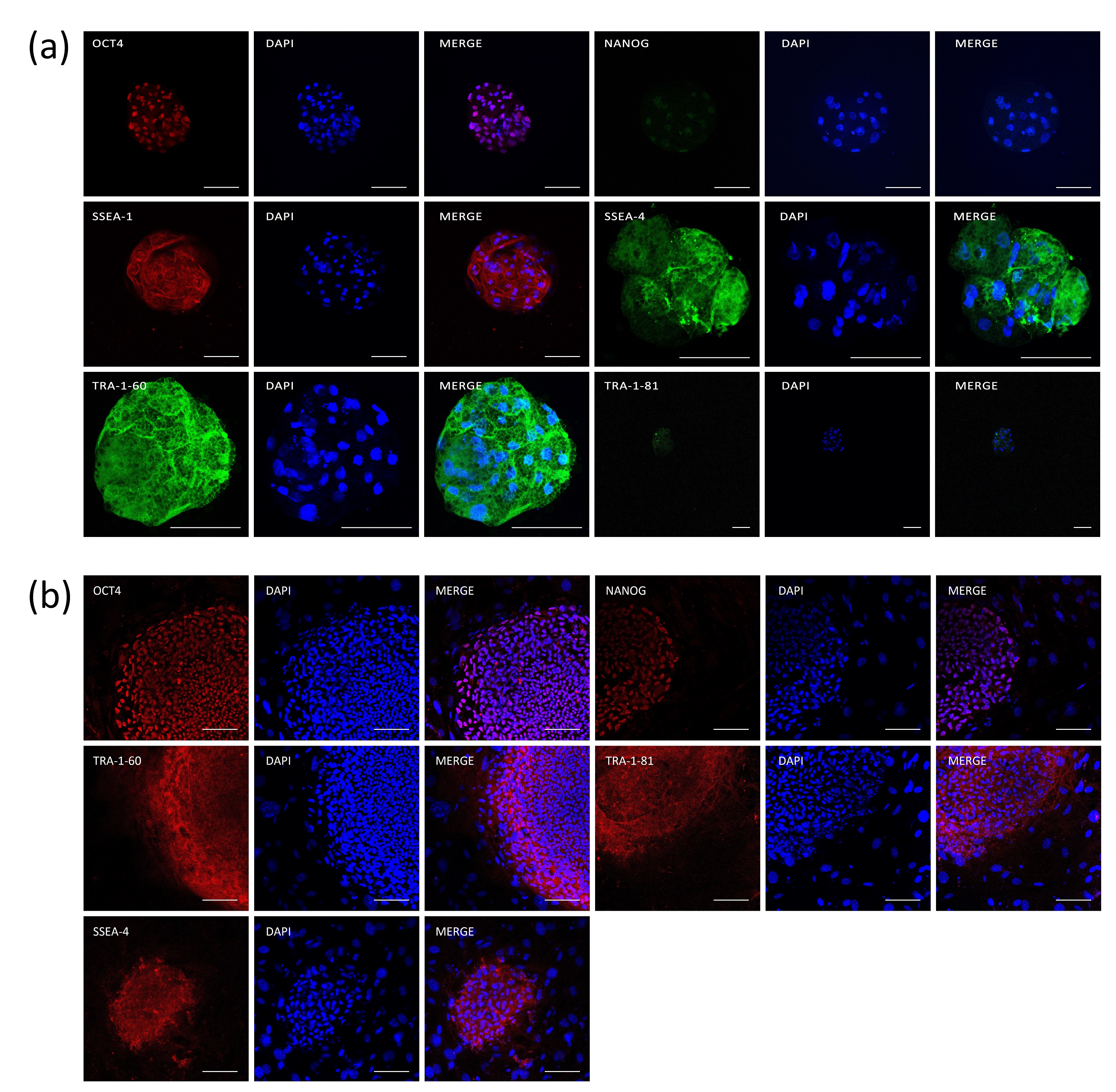
**

Immunofluorescence staining for pluripotency genes in pig day 7 blastocysts (a) and hES cells at passage 39 (b). Scale bar=100um.

**Supplementary Figure 2**

**
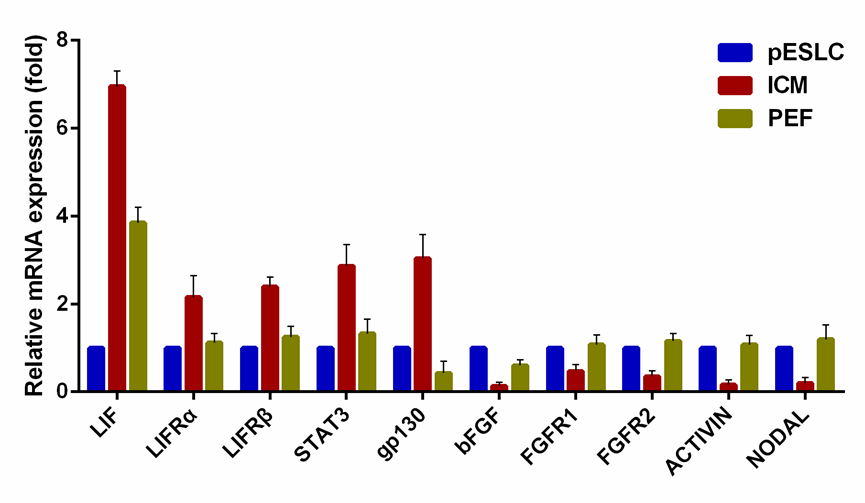
**

Relative expression of selected signal pathway genes at pESL cells, ICMs and PEFs. β-ACTIN was used as a housekeeping gene for normalization. Data is the mean ± SD.

**Supplementary Figure 3**


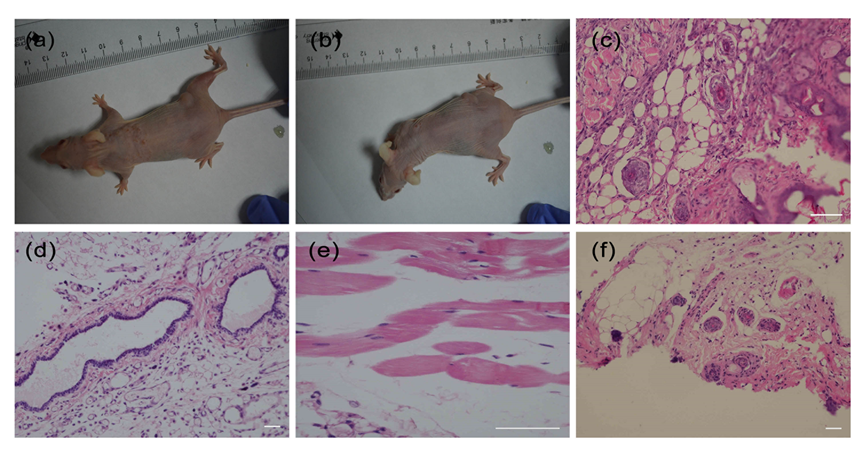


Teratoma formation from injected pESL cells is shown. (a,b) Gross images of the two mice showing smaller subcutaneous teratomas in the neck and dorsal flank regions. (c-f) Three germ layers present in teratoma derived from the pESL cell line. The images are H&E-stained sections showing representative endoderm (d, branched glands and duct), mesoderm (e, striated muscle and smooth muscle) and ectoderm lineage (c, f, neural rosette). Scale bar=100um.

**Supplementary Figure 4**


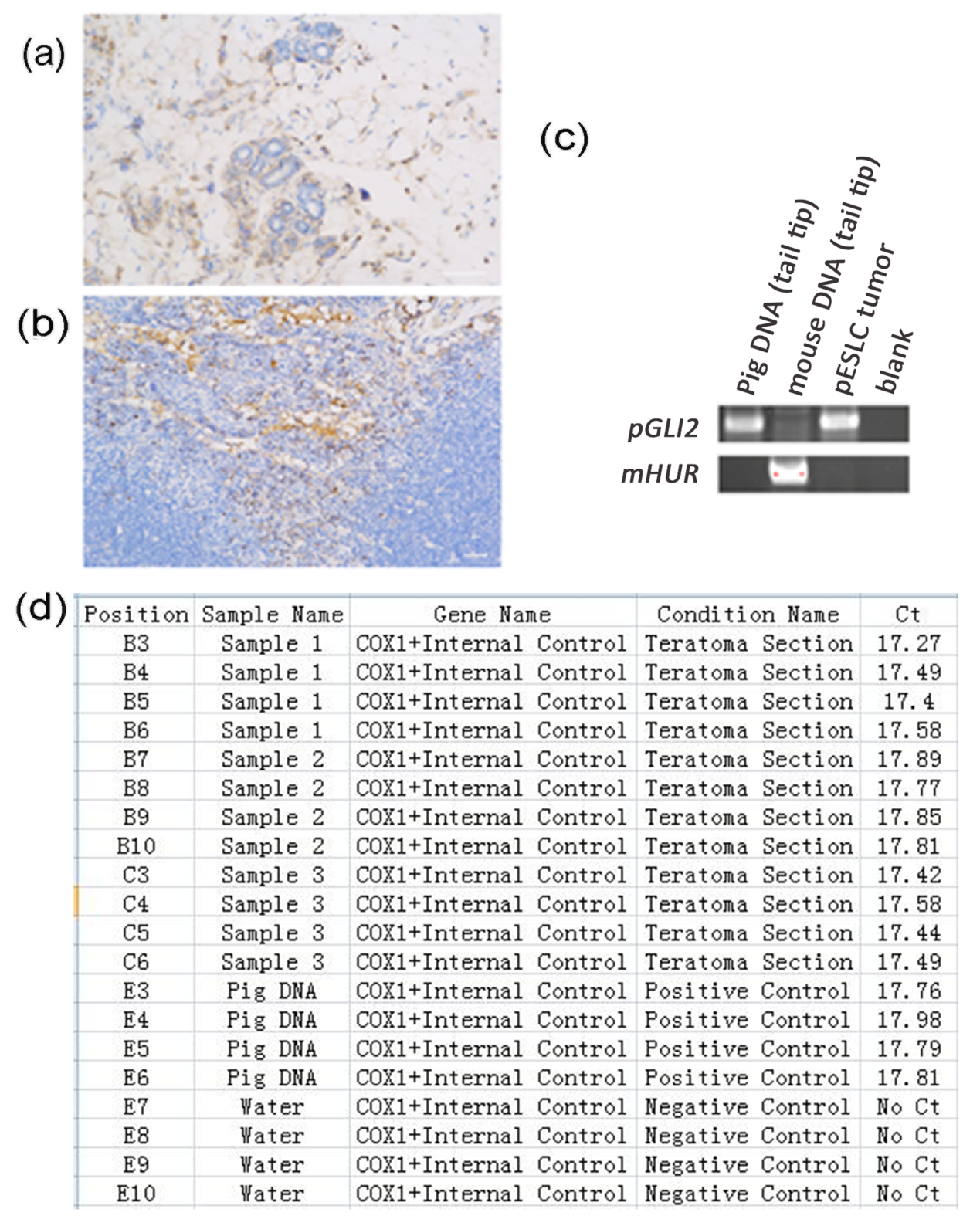


Identification of teratoma origin. (a, b) Pig IgM expression (brown) can be detected in paraffin-embedded teratoma sections by immunofluorescence staining. The cell nuclei (blue) was stained byhematoxylin. Scale bar=100um. (c) PCR analysis of genome from paraffin-embedded teratoma. Pig tissue (tail tip) and mouse tissue (tail tip) DNA were used to confirm pig specific origin of tumor tissue. The results reveal the expression of pGLI2 in tumor and pig tissue; and mHUR expression was detected only in the mouse tissue and not in tumor. blank: negative control. (d) Real-time PCR results of paraffin-embedded teratoma sections.

**Supplementary Tables**

**Supplementary Table 1. The Normalized Data of Microarray Analysis for Column Graph.**

**Supplementary Table 2. The Normalized Data of Microarray Analysis for** **Heatmap.**

**Supplementary Table 3. List of primers and conditions for PCR.**

| Gene | Primer sequence（5’-3’） | Accession number | Annealing  Temperature(℃) | Products  size（bp） |  |
| --- | --- | --- | --- | --- | --- |
| *OCT4* | F-AACGATCAAGCAGTGACTATTCG  R-GAGTACAGGGTGGTGAAGTGAGG | AJ251914 | 60 | 153 |  |
| *NANOG* | F-ATCCAGCTTGTCCCCAAAG  R-ATTTCATTCGCTGGTTCTGG | AY596464 | 60 | 438 |  |
| *SOX2* | F-GCCTGGGCGCCGAGTGGA  R-GGGCGAGCCGTTCATGTAGGTCTG | EU519824 | 66 | 443 |  |
| *c-MYC* | F-GCCAAAAGGTCGGAATCGGGG  R-CGCAGCACGTCTTTTTCTGACAC | X97040.1 | 60 | 358 |  |
| *LIN28* | F-AAACGCAGATCCAAAGGAGA  R-GCTCAATTCTGAGCCTCTGG | EU503118 | 54 | 386 |  |
| *KLF4* | F-AATGGTGTTGACCCCACCTTCTTC  R-TGAAAATGCCCGGTCGCACTTCTG | DQ000310 | 56 | 331 |  |
| *TBX3* | F-GGGACACTGGAAATGGCAGGAGA  R-AGGACGCGGGTGCTGGACGAGATAG | NC_010456.4 | 57 | 445 |  |
| *DPPA5* | F-CGGAGGTGTTCCAGGTCCAGACGC  R-GCATCCCTCGTTCCTGACGCTGGC | FJ436413 | 54 | 231 |  |
| *TFCP2L1* | F-GAAGATGTCCCGCGATGATCTGGT  R-CGTGGTAGCCGTCATTGCTTTCAG | NC_010457.4 | 66 | 401 |  |
| *REX1* | F-AGGCATTTTCTGGTTCATACTG  R-GTGAGGGGATTATTTTCAGTCTT | TC206552 | 56.5 | 248 |  |
| *CDX2* | F-GGAGCTGGAGAAGGAGTTTCA  R-TGCAACTTCTTCTTGTTGATTTTC | EU137688 | 58 | 153 |  |
| *MHCI* | F-CCTCTTCCTGCTGCTGTCG  R-AGCGTGTCCTTCCCCATCT | DQ992492 | 64 | 253 |  |
| *XIST* | F-GAAGCATCAGCCAGCAACAC  R-TCATAACCATCACTAGTACCCAAACC | AJ429140 | 58 | 182 |  |
| *AMYLASE* | F-GGAGAGATACCAACCAGTGA  R-ACCAGTTGGTGTTTAGGTTG | AF064742 | 52 | 488 |  |
| *ENOLASE* | F-GAGACAAATCTCGCTAGGTG  R-TGGAGACCACAGGATAGTTC | DQ676935 | 55 | 720 |  |
| *β-III TUBULIN* | F-CAGAGCAAGAACAGCAGCTACTT  R-GTGAACTCCATCTCGTCCATGCCCTC | DQ225361 | 56 | 250 |  |
| *GLI2* | F-GCACATCAACAACGAGCACATCCA  R-CATCCCATACGACCCATGTCACCC | NC_010457.4 | 54 | 205 |  |
| *mHUR* | F-GTTCCATGGCTCCCCATATC  R-AGCTTTGCAGATTCAACCTC | NC_000074.6 | 60 | 450 |  |
| *β-ACTIN* | F-GAGACCTTCAACACGCCG  R-GGAAGGTGGACAGCGAGG | U07786 | 52 | 685 |  |
